# Perceptions of health managers and professionals about mental health and primary care integration in Rio de Janeiro: a mixed methods study

**DOI:** 10.1186/s12913-016-1740-8

**Published:** 2016-09-30

**Authors:** Karen Athié, Alice Lopes do Amaral Menezes, Angela Machado da Silva, Monica Campos, Pedro Gabriel Delgado, Sandra Fortes, Christopher Dowrick

**Affiliations:** 1 Programa de Pós Graduação em Ciências Médicas/ Faculdade de Ciências Médicas, Universidade do Estado do Rio de Janeiro, Rio de Janeiro, Brazil; 2 Laboratório Interdisciplinar em Atenção Primária à Saúde/LIPAPS/Faculdade de Ciências Médicas, Universidade do Estado do Rio de Janeiro, Rio de Janeiro, Brazil; 3 Escola Nacional de Saúde Pública, Fundação Oswaldo Cruz, Rio de Janeiro, Brazil; 4 Núcleo de Pesquisas em Políticas Públicas de Saúde Mental/ Instituto de Psiquiatria da Universidade do Brasil, Universidade Federal do Rio de Janeiro, Rio de Janeiro, Brazil; 5 Institute of Psychology Health and Society, University of Liverpool, Liverpool, UK

**Keywords:** Mental health, Primary care, Collaborative care, Mental health matrix support, Integration, LAMIC, Mixed methods, MHGAP, Public health, Implementation science

## Abstract

**Background:**

Community-based primary mental health care is recommended in low and middle-income countries. The Brazilian Health System has been restructuring primary care by expanding its Family Health Strategy. Due to mental health problems, psychosocial vulnerability and accessibility, Matrix Support teams are being set up to broaden the professional scope of primary care. This paper aims to analyse the perceptions of health professionals and managers about the integration of primary care and mental health.

**Method:**

In this mixed-method study 18 health managers and 24 professionals were interviewed from different primary and mental health care services in Rio de Janeiro. A semi-structured survey was conducted with 185 closed questions ranging from 1 to 5 and one open-ended question, to evaluate: access, gateway, trust, family focus, primary mental health interventions, mental health records, mental health problems, team collaboration, integration with community resources and primary mental health education. Two comparisons were made: health managers and professionals’ (Mann-Whitney non-parametric test) and health managers’ perceptions (Kruskall-Wallis non parametric-test) in 4 service designs (General Traditional Outpatients, Mental Health Specialised Outpatients, Psychosocial Community Centre and Family Health Strategy)(SPSS version 17.0). Qualitative data were subjected to Framework Analysis.

**Results:**

Firstly, health managers and professionals’ perceptions converged in all components, except the health record system. Secondly, managers’ perceptions in traditional services contrasted with managers’ perceptions in community-based services in components such as mental health interventions and team collaboration, and converged in gateway, trust, record system and primary mental health education. Qualitative data revealed an acceptance of mental health and primary care integration, but a lack of communication between institutions. The Mixed Method demonstrated that interviewees consider mental health and primary care integration as a requirement of the system, while their perceptions and the model of work produced by the institutional culture are inextricably linked.

**Conclusion:**

There is a gap between health managers’ and professionals’ understanding of community-based primary mental health care. The integration of different processes of work entails both rethinking workforce actions and institutional support to help make changes.

**Electronic supplementary material:**

The online version of this article (doi:10.1186/s12913-016-1740-8) contains supplementary material, which is available to authorized users.

## Background

Mental health problems [1] are a challenge to public health systems, especially in low and middle-income countries (LAMIC) [2, 3]. Studies [4, 5] have demonstrated that they are associated with important economic and social problems regarding global health and the impact of sustainable development [6]. Primary health care has been understood as the basis for reducing the gap between population needs and care offers, especially delivered through primary care and communitarian actions [7, 8]. Integration and delivery of primary mental health care are of increasing importance for the achievement of these goals.

The literature review on studies about primary care and mental health integration covers a broad scope including Human Rights [9], Welfare State organisation [10], the integration of different work processes [11–13], human resources training [14], access [15], common mental disorders [16, 17], medication [18] and psychosocial interventions [17]. Integrating health actions is complex and health managers and care professionals need more than just to agree to share the same physical place in order to work together [19, 20]. Therefore, this complex practice emphasises how important it is to prepare human resources to deliver mental health care in the community [1] and to organise integrated work processes, both of which are considered challenges to this integration [21].

Furthermore, little is known about professionals’ perceptions regarding integrated care. A single study in Latin America that focused on health professionals’ [22] opinions, suggests that managers’ and health professionals’ opinions are based on past experience, expectations, definitions of quality of care and power relationships between health professionals. A second point is a wide-ranging and confused terminology concerning primary mental health, which can also be a barrier to defining collaboration processes between different workers [23]; although the words used to define them can be very similar, interpretations on how to apply them can vary very often. the way to interpret and apply them is variable.

Additionally, as integrated care is an international recommendation requiring changes in the organisation of health systems, especially in LAMIC, these changes are being made all over the world, including Brazil. The SUS (the Brazilian National Health Service, or literally the Unified Health System)^1^, created with the new constitution in 1988 during the country’s redemocratisation process, is based on the tenets of universal, integral and equal rights of access to health. Based on these principles, changes to strengthen community-based primary care services have been implemented since the 1990s. In Brazil, traditionally, primary care was organised into General Traditional Outpatient services (GTO). These units covered large geographical areas, with 100,000 inhabitants, involving professionals from different medical backgrounds in basic specialities. After 1994, this primary care model started to change progressively to the Family Health Strategy (FHS) model, where multidisciplinary teams are responsible for 3500 people (not necessarily patients) living in a community-ascribed area [24] (see Table 1).

**Table 1:** SUS primary care and mental health services

Assistance model design	Traditional model (Currently being deactivated)		Primary care model (Currently being implemented)		
Care focus	Individual and disease-oriented		Health, community and territory-oriented		
Kind of Service	General Traditional Outpatient Service (GTO)	Mental Health Outpatient Service (MHOS)	Family Health Strategy (FHS)	Mental Health Matrix Support Teams (MHMS)	Psychosocial Community Centre (PCC)
Care Level	Primary	Specialised	Primary	Primary	Specialised for severe mental health patients
Coverage	100,000 inhabitants from a geographical area.	This service is not commonly associated to patient’s territory.	3500 enrolled people in delimited territory.	Each team covers up to 9 FHS teams, according to population size.	100,000 to 200,000 inhabitants from a defined geographical area, which depends on patient’s territory.
Access Design	Consultations are booked on demand.	Consultations are booked on referral from another health professional.	Consultations and care are delivered in units located in the community. The evaluation focus considers not only the individual but also family and community context.	Provides support to FHS teams and works in collaboration, assisting their patients.	Consultations are booked either by referral or on demand.
Team Composition	Internal medicine, paediatrics and gynaecology outpatient clinics provide general care. Frequently, there is also a mental health clinic.	Psychiatrists and psychologists.	Multidisciplinary team comprise: 1 family physician, 1 nurse, 1 nursing technician and 6 community health workers. Perform active search of patients. Work according to primary care premises: gateway, longitudinally, comprehensiveness and care coordination.	Multidisciplinary team composed of professionals, including one mental health professional (e.g. a psychologist or a psychiatrist). Health managers define the team based on epidemiological data, local needs and the number of health teams to be supported.	Multidisciplinary team consisting of: neurologists, nurses, nursing technicians, pharmacists, nutritionists and psychiatrists, psychologists, social workers, speech therapists, music therapists, occupational therapists, among other multi non-specialised professionals admitted to the team.
Mental Health Clinical and Assistance offers	Referred patients with mental health problems to a mental health outpatient clinic.	Specialty consultations based on referral and counter-referral proceedings. Their routine does not include working with primary care professionals.	When patients with mental health problems are identified, FHS requests the support of MHMS Teams and works in collaboration with them to provide mental health care in the community.	MHMS works at least once a month with FHS teams in order to improve the FHS teams capacity to identify emotional suffering and take care of it and monitor mental health cases. This includes integrating care actions delivering mental health care in the territory.	Provide care to patients with severe mental problems. PCC works with FHS under two circumstances: either referring their own patients to FHS teams or helping FHS teams provide care to severe mental health patients already being treated by the FHS teams.

This change in Brazilian Primary Care, delivering health services in communities instead of waiting for populations’ demands, brought about important improvements in public health indicators, such as an increase in the detection of neglected tropical diseases, and reduced health disparities and child mortality. The communitarian basis revealed a high prevalence of mental health problems in the ascribed population, which is associated with psychosocial problems, lower quality of life and clinical co-morbidities [25, 26]. Due to SUS’ equal tenet, FHS teams have been initially implemented in low-income areas where they must deal with problems such as domestic violence and drug dealers, as well as treating diseases such as diabetes, HIV and hypertension.

**Fig. 1:**
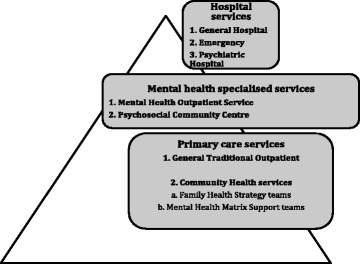
The SUS services including mental health offers

In parallel, also since the 90’s, mental health services started to undergo reform shifting from long-term Psychiatric Hospital beds to Mental Health Psychosocial Community Centres (PCC). This is the target of the Brazilian Psychiatric Reform [27], which set out the Brazilian Mental Health Policy in 2001. PCC were created to care for those patients with severe mental health problems within a population of 100,000 inhabitants. For patients with less severe mental disorders, but still in need of specialised mental health care, there are Mental Health Outpatient Services (MHOS). Their integration is by referral and counter-referral processes (Table 1). Moreover, in view of the stigma surrounding mental health issues and the vulnerability of the low-income population covered by FHS, a local model of collaborative care was developed, based on Mental Health Matrix Support teams (MHMS). These teams help FHS teams to deal with psychosocial problems and common mental disorders. Collaborative care entails continuing education, which involves enabling capacity building of the FHS teams while MHMS teams assist them organising the delivery of mental health care in communities as well as helping them in the treatment of traditional primary mental health problems, such as medically unexplained symptoms, psychosomatic problems and common mental disorders (see Fig. 1).

One of the main problems faced by SUS is the integration of different service levels, which aims to increase access to the most vulnerable population living in very low development conditions [24], and narrow the gap between mental health needs and treatment offer. Therefore, the Brazilian Mental Health Service is divided into three levels: primary care services, specialised outpatient units and hospitals, as shown in Fig. 1.

**Fig. 2:**
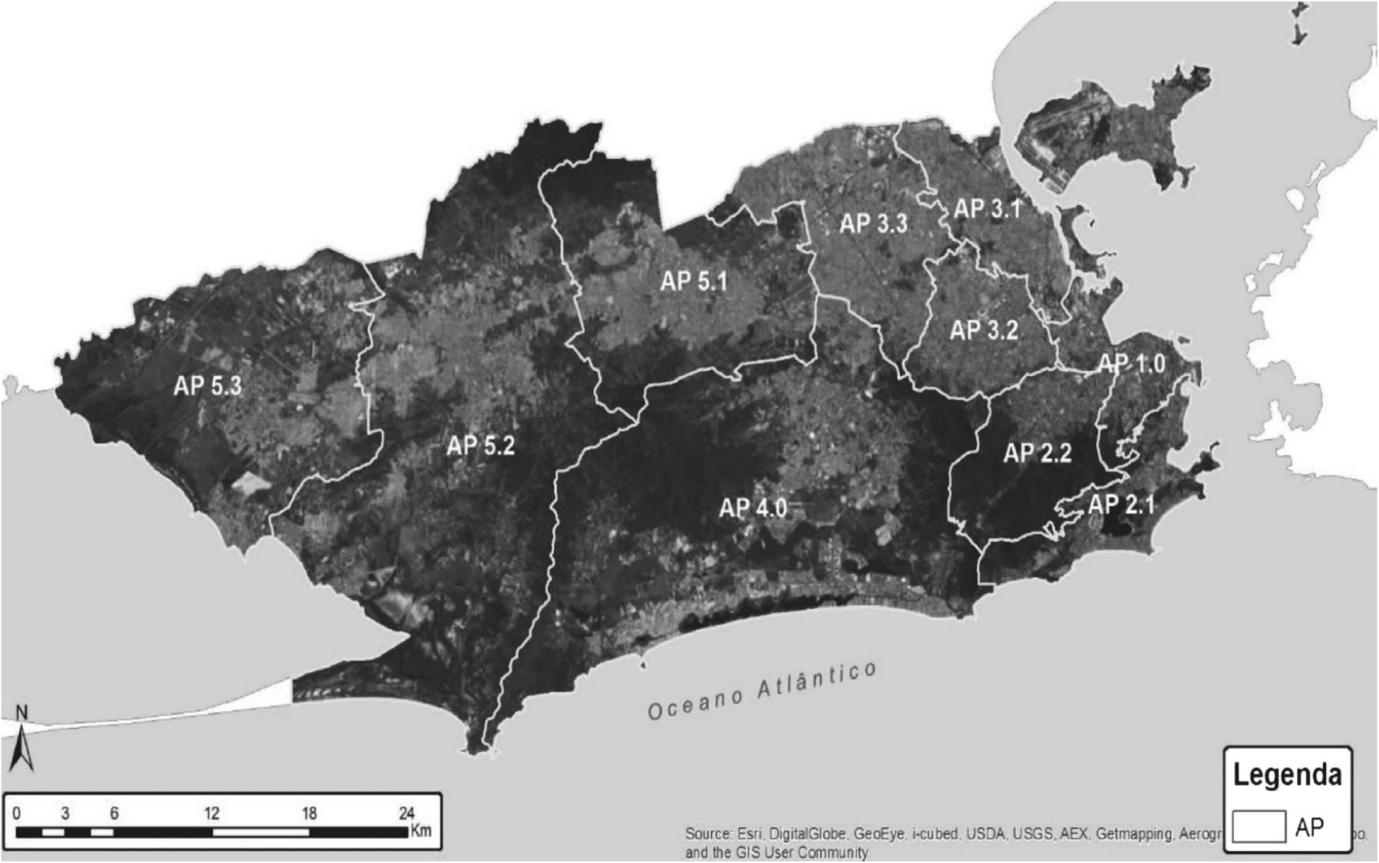
Rio de Janeiro’s programmatic areas [42] (AP = Área Programática/Programmatic Area)

Rio de Janeiro City has been an important example of this process, with a shift from General Traditional Outpatients to FHS teams, increasing their coverage from 3.5 % in 2008 to 40 % in 2012 [28, 29], mainly in poor and violent inner-city communities [30] (Fig. 2).

The context includes important social contrasts, notably between favelas (slums) and middle and upper-class homes, which coexist in the same neighbourhood [31, 32]. Thus, the Human Development Index varies from high to very low (e.g. 0.97 in Gávea neighbourhood vs. 0.25 in Realengo) [33–36]. There are stark differences even in poor areas. For example, in total, there are about 800 favelas and their social development index ranges from 0.63 (medium) to 0.25 (very low). Over 750 of those are below 0.59 (low human development). Additionally, some favelas, often controlled by drug dealers [37], tend to be violent in spite of political and military actions implemented since 2010.

Preferentially, primary care units have been located in places accessible to these vulnerable communities, which in Rio are usually found on the hillsides next to middle-class neighbourhoods.

Hence, in 2008, considering collaborative care experiences from the United Kingdom and other countries [38–40], a research group^2^ on mental health in primary care from the University of the State of Rio de Janeiro (UERJ) started to work with primary care teams in one of Rio de Janeiro’s Health Districts, known as Programmatic Area 2.2, with 371,120 inhabitants.

Due to patients’ problems such as anxiety, depression, post-traumatic stress and unexplained medical complaints, a group of researchers, professors, residents and health professionals undertook actions aimed at mental health and primary care professionals working together. These actions were based on the integration of mental health and primary care in this part of Rio and included training primary mental health workers. Thus, a partnership involving the University of the State of Rio de Janeiro(UERJ) and the coordination staff of Programmatic Area 2.2 has fostered MHMS activities in the health units in this region with a view to integrating mental health teams and primary care.

These collaborative experiences between the local services and UERJ tested this integrated model of work (MHMS) while national deliberations (Law 154/2008) on an interdisciplinary collaborative care model for SUS were approved. The Matrix Support Teams, officially called NASF (Núcleo de Apoio a Saúde da Família), were created to work together with FHS in order to improve their capacity. So, the matrix support implemented in PA 2.2, at this historical moment, can be considered an innovation involving all primary care services, outpatient services and psychosocial community centres. When this research was carried out (between 2011 and 2013), PA 2.2 had not completely deployed the NASF team based on the national model references. This pioneer experience in PA 2.2 was important also to develop the first practical national recommendations about mental health matrix support for the NASFs, the Practical Guide to Primary Mental Health Care [41].

The purpose of this paper is to study health managers’ and professionals’ perceptions, in their different services, about the integration of mental health in primary care settings. The main goal is to analyse them and point out key messages related to planning, organisation and implementation of collaborative care to reduce the local mental health gap and foster continuity of care.

## Method

### Design of the study

This mixed-method cross-sectional study was developed to map different aspects of mental health and primary care integration from the perspective of health managers and health professionals. Thereby, considering them as potential supporters or stakeholders of the integration of mental health in primary care, this research has tackled three research issues. The quantitative study pointed out how they evaluate their health actions regarding primary mental health care references, and the qualitative one, as a necessary complement, explored their experience of MHMS.

The issues were:

- health managers’ and professionals’ perceptions about primary mental health care implementation in Rio in the period studied;
- the similarities and differences between health managers’ and professionals’ perceptions;
- prospects for Brazilian MHMS.

### Setting characteristics

#### Geographical and socio-economic context in Rio de Janeiro’s favelas

This study was conducted in Rio de Janeiro City, which is divided into ten Health Districts (Programmatic Areas) (Fig. 2) [42], all of them providing similar mental health and primary care services. This programmatic area (PA 2.2) has 371,120 inhabitants in 7 neighbourhoods: Alto da Boa Vista, Praça da Bandeira, Tijuca, Andaraí, Grajau, Maracanã and Vila Isabel. For public health management purposes, each neighbourhood is divided into micro-areas. Although the area’s Human Development Index (HDI) is about 0.9, which is similar to rich countries, the region is marked by social contrasts. According to the last national census, monthly income ranged from $ 61.12 to 3558.59 [34] (American dollars). The Social Development Index (SDI) verified that the highest rate is 0.7 in Maracanã (high development conditions) and the lowest is 0.54 in Praça da Bandeira (low development conditions). The favela population accounts for around 43,000 inhabitants. In the micro-areas of this study the ratio ranges from 0.55 (low) in Parque Vila Isabel to 0.48 (very low) in the Casa Branca Community. Moreover, 4 of the 37 Peacemaking Police Units are located in this region. Since 2010, these units have been deployed in Rio de Janeiro’s favelas and other underprivileged areas with violence and drug dealing.

#### SUS Primary Care and Mental Health Services Designs in Programmatic Area 2.2

The survey was conducted with health professionals working in all primary care and mental health units in this region: General Traditional Outpatient Services (GTO)(*n* = 4), Mental Health Outpatient Services (MHOS)(*n* = 4), Psychosocial Community Centres (PCC)(*n* = 2), Family Health Strategy (FHS)(*n* = 8). In view of the complex makeup of the SUS (Fig. 1), with part of the services being deactivated and another part being implemented, some important aspects of these services must be highlighted, such as care focus, service status, care level, coverage, access design, team composition, principles of mental health clinical and assistance management (Table 1).

### Participants

In this research all professionals involved in mental health matrix support activities in PA 2.2 were interviewed, including family health doctors and MHMS supporters, as well as all health unit managers in this area. Due to the moment of this new model’s implementation in this area, the group concerned was still small. Thus, in total, all 42 people working with MHMS were interviewed (35 female and 7 male), covering all health managers (15 female and 3 male), general practitioners, psychologists and psychiatrists (20 female and 4 male), all involved in primary care and mental health integration in the territory studied.

Other FHS professionals, such as nurses and community health workers were excluded. The reasons were: firstly, during the period studied, MHMS activities were mainly between doctors and mental health professionals, and secondly, despite the relevance of other FHS professionals’opinions, this project had financial constraints and, consequently, nine more nurses and fifty-four Community Health Workers, who did not take active part in the mental health matrix support work, were not interviewed.

Among the 18 health managers we observed different professional backgrounds, namely: doctors (8), nurses (6), dentist (1), physiotherapist (1), social worker (1), psychologist (1) and of the 24 health professionals, there were General Practitioners (13), psychiatrists (4) and psychologists (7).

After obtaining approval from an official local district council and from the university’s ethical committee, these professionals were invited either by email or phone and asked to participate voluntarily. All of them signed an informed written consent approved by the ethical committee. Each interview took approximately 90 min; all of them were recorded.

### Data collection tool: creating an instrument to evaluate mental health and primary care integration

A semi-structured questionnaire (see Additional file 1) was created to quantitatively evaluate these professionals’ perceptions of their own services in relation to integration processes, followed by one open-ended question inquiring about positive and negative aspects of MHMS. To create this instrument, three other instruments were considered. The first is an international reference created to evaluate primary care services, The Primary Care Assessment TOOL [43, 44]; the second is the corresponding Brazilian version of the first, adapted to the Brazilian context and validated into Brazilian Portuguese [45]; the third is a questionnaire to evaluate Brazilian multidisciplinary teams [46].

The questionnaire, specially developed for evaluating primary care and mental health integration, comprised 185 questions, divided into different sections (Table 2). Alternatives were presented on a Likert scale ranging from 1 (none or never), 2 (almost never), 3(sometimes), 4 (almost always) to 5 (always). In the mental health problems section, which sought to identify how professionals observed specific mental health problems among patients coming into their units, the scale varied from 0 (no-one), 1 (low frequency), 2 (medium frequency), to 3 (high frequency). Besides the answer scale, the section about mental health interventions and integration with the community had space for freely stating examples. At the end of the survey, there was an open-ended question about positive and negative aspects of primary care and mental health integration, related to the previous 6 months.

**Table 2:** Description of questionnaire’s sections

Section	Objective
General Information	To map profession, function and workplace.
Access	To map whether patients can access services, medicines and consultations.
Gateway	To map the different services available to patients in the unit
Trust	To map the relationship between patients and health professionals, and patients and units
Primary Mental Health Interventions	To map psychosocial actions offered.
Primary Mental Health Record	To map if mental health interventions are recorded in the Health Record System as mental health interventions or primary care interventions.
Collaboration between Teams	To map collaborative work with different health teams and services as well as with other institutions such as health services, schools or community services.
Mental Health Problems	To map mental health problems treated in the unit.
Family Focus	To map family interventions.
Integration with Community Resources	To map institutional integration with community resources.
Primary Mental Health Education	To map educational expectations regarding mental health in primary care.
Positive and Negative Aspects (Open-ended question)	To map positive and negative aspects of primary care and mental health integration.

#### Pilot study of mental health and primary care integration instrument

A pilot test was conducted in two stages. Firstly, undergraduate students training to work in primary mental care settings applied the questionnaire to verify language problems and the amount of time needed to answer each question. The problems were adjusted to improve the instrument, which was also discussed individually and collectively with mental health and primary care professionals and managers from other territories. Secondly, each of the three research applicants made one training interview with a health professional working outside PA 2.2.

#### Periods of interviews

The interviews occurred in two periods. Firstly, managers were interviewed from September 2011 to January 2012. The health professionals’ interviews were collected from December 2012 to November 2013. From this group one GP did not participate as he was on holiday. Due to the lack of financial resources to cover researchers’ interview fees, the second period was longer than planned.

#### Alpha crombach

In order to validate the questionnaire, Cronbach’s Alpha was calculated, after concluding the managers’ interviews, so that internal consistency could be evaluated. The internal consistency by section was: unacceptable for Access (*a* = 0.495); questionable for Gateway (*a* = 0.649) and Integration with Community Resources (*a* = 0.662); acceptable for Trust (*a* = 0.729), Collaboration between Teams (*a* = 0.764) and Mental Health Problems (*a* = 0.762); good for Family Focus (*a* = 0.852); and excellent for Primary Mental Health Interventions (0.909), Mental Health Interventions Record (*a* = 0.912) and Primary Mental Health Education (*a* = 0.882). The final result was 0.730 (*p*-value 0.000), which was considered acceptable [47–49].

### Analysis

Analysis of the database involved two interpretative phases. First, while the quantitative analysis compared 1) health managers considering their design services and 2) health managers’ and professionals’ perceptions as a workforce, the qualitative analysis considered the whole group’s perceptions about the advantages and disadvantages of MHMS. Secondly, to answer the three key issues about mental health and primary care integration, a framework was created to identify convergences and divergences between qualitative and quantitative results and associate them.

#### Quantitative analysis

Data were analysed with SPSS statistical software (version 17.0). The Mann-Whitney non-parametric test was used to compare health professionals’ and managers’ perceptions. The second comparison was to observe different types of primary care and mental health managers’ perceptions. The Kruskall-Wallis non-parametric-test was used to compare managers’ perceptions in 4 kinds of services (GTO, MHOS, PCC and FHS). Numerical data were calculated based on the number of answers to each section. The missing values were not excluded; they were replaced by mean values.

Considering each dimension studied, the hypotheses were:

- Hypothesis 1. Health managers and professionals diverge in their answers evaluating mental health and primary care integration.
- Hypothesis 2. Managers from different services diverge in their answers evaluating mental health and primary care integration.

#### Qualitative analysis

Examination was based on framework analysis and thematic analysis methods.

Qualitative data encompassed all information that participants were asked to provide to complement or exemplify their answers to the quantitative survey questions. For instance: descriptions of resources; examples of mental health interventions not mentioned in the questionnaire; examples of collaboration between health services, education and community, and of integration with other services. Besides that, an open-ended question requested explanations about positive and negative aspects of mental health and primary care integration.

The content was described and organised according to the framework analysis method [50], which allowed for the comparison of positive and negative aspects. Firstly, content analysis enabled grouping of a large amount of text into categories. This analysis was processed using the same quantitative method dimensions (Table 2) as a filter. To interpret the data, interviews were repeatedly read and listened to and ideas about key aspects were noted down to develop initial codes as a thematic analysis. Next, they were linked according to their nature and contents to length and frequency of these perceptions [24, 51]. These codes helped to arrange the data into broader themes, which were revised. The discussion between three researchers processed the data into the most prominent ideas. These ideas were organised into three categories. Although this field of study is treated in interesting international studies [52, 53], this approach was chosen due to lack of past research in this field, where the growth of MHMS teams in SUS’s structure is being progressively implemented in order to cover a country with more than 5000 municipalities. Thus, themes were developed inductively [54].

#### Mixed method

The integration [55, 56] of these two methods was arranged in a framework considering three key issues introduced at the beginning of the method section. Subsequently, qualitative and quantitative answers were compared to see if they converged or diverged. The analysis was conducted according to primary mental health care references [57].

## Results

### Quantitative results

#### Health Managers’ and Professionals’ perceptions

Given that health managers and professionals diverge, their perceptions about the results of different points evaluated in this study proved to be similar in most respects.

The group evaluated Access (2.9/5) worse than Gateway (3.5/5), which means that while about 60 % of the population they are covering can have access to their services, once this population arrived at this offer this sample considered that people have more chances (70 %) of starting a treatment. Trust (4.3/5) demonstrated that this group evaluated their relationship as positive with people that they take care of. Mental Health Interventions (2.8/5) highlighted that this sample does not consider that Mental Health interventions are offered to a percentage smaller than about 55 % of their patient needs. Team Collaboration (3.3/5) showed that this group as a whole has a positive perception of their co-joint actions. However, their evaluation of the record system revealed different perceptions between the two groups included in this sample. While assistance professionals evaluated (2.4/5) that less than 50 % of these recorders are adequate, health managers had a more positive view of this point, considering (3.3/5) about 70 % of these recorders adequate. Mental Health Problems (1.6/4) highlighted that the perceptions of the interviewees were 40 % between a low (1 to 30 %) and a medium (31 to 60 %) frequency of mental health problems in the patients treated in their units. Family Focus (3.4/5) results showed that they usually work considering family problems in about 70 % of cases. However, in Integration with Community Resources (2.6/5), they demonstrated that they work with these resources in about 50 % of their needs. Concluding, Primary Mental Health Education (3.4/5) highlighted that continuing educational activities to prepare interviewees and their unit teams to work with primary mental health care is positive in 70 % of the answers (Table 3).

**Table 3:** Comparing health managers'and professionals' perceptions

Health Managers’ and Professionals’ perceptions Mann-Whitney non-parametric test (M-W)	Mean			Std. deviation		
	Health Managers and Professionals (*n* = 42)			Health Managers and Professionals AP		
	Prof. *n* = 24	Man. *n* = 18	Total	Prof.	Man	Total
Access	2.9	3.0	2.9	.64	.43	.55
Gateway	3.5	3.6	3.5	.71	.74	.72
Trust	4.3	4.4	4.3	.37	.37	.37
Mental Health Interventions	2.8	2.8	2.8	.38	.63	.50
Is Record System adequate to register mental health actions?*	2.4	3.3	2.8	1.12	1.60	1.41
Collaboration between Teams	3.2	3.3	3.3	.52	.57	.54
Mental Health Problems	1.6	1.5	1.6	.38	.21	.32
Family Focus	3.4	3.3	3.4	.66	.96	.79
Integration with Community Resources	2.5	2.7	2.6	.76	.59	.69
Primary Mental Health Education	3.4	3.4	3.4	.90	1.08	.97

*M-W test was performed with *p*-value < 5 %

#### Managers’ perceptions of service design

Concerning the hypothesis that managers from services diverge in their evaluation of mental health and primary care integration in their own routine, the findings confirmed that units see things differently in this regard.

Access dimension (*p*-value < 5) highlighted that managers working in mental health outpatient services consider that only about 50 % (2.4/5) of people that need their services have access to them. The other managers evaluated this higher, (in about 65 % (3.2/5) of cases, people have access to their services). Managers perceived Gateway similarly: about 70 % of patients (3,6/5) are treated in these services when they have access to them. Trust (between patients and health professionals) was the best evaluated section (4.4/5), which means that these managers considered that their relationship with patients is good. In Mental Health Interventions (*p*-value < 5), non-specialised (GTO = 2.1; FHS = 2.8) services evaluated their actions worse in terms of mental health activities than specialized services (MHOS = 3.0; PCC = 3.8); Record System was considered adequate in about 70 % of cases (3,3/5). In Collaboration between Teams, GTO(2,6/5) managers made a worse evaluation, followed by MHOS(3,3), FHS(3,6) and PCC(3,7). Mental Health Problems (1.5/4) highlighted that interviewees’ perceptions were 37 % between a low (1 to 30 %) and a medium (31 to 60 %) frequency of mental health problems in the patients treated in their units. Family Focus is best evaluated in services based in communities. While GTO(2,3) and MHOS(2.8) evaluated that their offer has a Family Focus in less than 60 % of their cases, PCC(4.0) and FHS(3.9) evaluated that they offer that in 80 % of cases. Integration with Community Resources (2.7/5) are smaller than 60 %. To conclude, Primary Mental Health Education (*p*-value < 5) is better evaluated in community-based services (PCC = 4,7;FHS = 3,9), followed by MHOS(3.0/5) and GTO(2.3/5), which evaluated this point as very low.

Especially noteworthy was the following contrast: Mental Health Interventions, Collaboration between Teams; Family Focus and Primary Mental Health Education received the worst evaluation by the General Traditional Outpatients and the best by the Psychosocial Community Centre. In Team Collaboration, Psychosocial Community Centre as specialised services and FHS as non-specialised services obtained very similar values (3,6/5), which means that they considered they work collaboratively in about 70 % of their cases; likewise, for Family Focus (3,9/5), which means they believe they usually care for people using this strategy in about 80 % of their cases (Table 4).

**Table 4:** Comparing different managers' perceptions perspectives

Managers’ perception Kruskall-Wallis non-parametric test (K-W)	Mean					Std. deviation				
	Services (Managers answers/4 groups)					Services				
	GTO *n* = 4	MHOS *n* = 4	PCC *n* = 2	FHS *n* = 8	Total *n* = 18	GTO	MHOS	PCC	FHS	Total
Access*	3.1	2.4	3.2	3.2	3.0	.31	.42	.18	.34	.43
Gateway	3.5	3.5	3.2	3.8	3.6	.89	1.11	.27	.59	.74
Trust	4.2	4.4	4.5	4.5	4.4	.28	.24	.44	.46	.37
Mental health Interventions*	2.1	3.0	3.8	2.8	2.8	.37	.56	.05	.48	.63
Is Record System adequate to register mental health actions?	3.3	4.4	4.0	2.5	3.3	1.52	.42	.47	1.91	1.60
Collaboration between Teams*	2.6	3.3	3.7	3.6	3.3	.37	.47	.49	.39	.57
Mental Health Problems*	1.3	1.5	1.8	1.6	1.5	.17	.11	.33	.17	.21
Family Focus*	2.3	2.8	4.0	3.9	3.3	.63	.35	.00	.86	.96
Integration with Community Resources	2.6	2.6	3.0	2.8	2.7	.43	.94	.24	.58	.59
Primary Mental health Education*	2.3	3.0	4.7	3.9	3.4	1.20	.72	.47	.71	1.08

*K-W test was performed with *p*-value < 5 %

### Qualitative results

#### Descriptive information: structure, examples of interventions and partnerships

Some questions had space for examples or specific information. The answers that appeared in this study are:

1. Analysis of contact resources showed that all units have an e-mail, telephone and access to the Internet.
2. Regarding Mental Health Interventions, the examples shared indicated in general a large provision of groups for patients with different health conditions, such as: chronic diseases; reducing health problems; family planning; pregnancy; adolescents; the elderly; anti-smoking; alcoholics anonymous; handicrafts; gardening; income generation; yoga and massage.
3. Regarding Integration with Community Resources, professionals mentioned as examples: outpatient units; emergency units; hospitals; university services; programmatic area coordination; health system information; nurseries; schools; “Health in the School” program; police; shelters for women, men and the elderly; non-profit organisations; football teams; samba schools; community radio and Peacemaking units.

#### Open-ended question: positive and negative perceptions of mhms

The open-ended question was to elicit from the interviewee their opinions about mental health and primary care integration regarding MHMS actions. Thus, these professionals were asked to share their positive and negative opinions. As the managers sample was very small, this research group analysed this answer without comparing health managers and professionals.

As a whole, the outcomes highlighted significant aspects of MHMS organisation, workforce and practice. Taking into account the different analytical themes, health professionals’ perceptions were organised into three categories: Network, Primary Mental Health Education and Primary Mental Health Interventions. Communication problems emerged as a common issue in all situations related to primary care and mental health integration and they are present in all three categories.

The Network category considered how the structural components of MHMS teams are connected, including logistics, human resources, workforce, and organisational partnerships. This category highlighted issues such as networking, integration and institutional communication. For instance:

*“The municipal government should constantly communicate the workflow between specialists and non-specialists because we do not know where new FHS teams are being created, what their scope is and which territory they cover”. (Manager 1, outpatient specialized services psychiatrist).*

This quotation illustrates the lack of knowledge about different systems’ structures and how that can negatively affect the interactions between different services. On the other hand, the quotation below demonstrates how positive the network can be when the partnership between services is clear.

*(…) when we have this strong partnership, we access a mental health support team, doing referrals and counter referrals, leading to better understanding of the case for planning care”. (Manager 1)*

The Primary Mental Health Education category referred to the relevance of integrating knowledge and expertise between different workforces. Not only is it suggested that health professionals need to learn from each other, but also that different work processes must be integrated and further knowledge may be acquired by all the professionals involved. For example:

*“(…) patients already exist, but we need (****as primary care professionals****) to better understand what it is possible to do in order to offer support to them here, because it is so difficult to work with mental health problems.”* (Manager 2, FHS, nurse)

This quotation highlighted how difficult it is for a non-specialist to care for mental health problems without tools or enough knowledge to tackle these problems, including the capacity to recognise mental health problems. Similarly, this same quotation showed that the professional considers it important to receive technical support to be able to help patients with this type of problem.

“*The problem is the lack of human resources, because all that****(regarding the health system changes in the city)****will raise demand, and unfortunately we cannot address current problems”. (Manager 3, GTO, doctor)*

Concerning Primary Mental Health Education, while the previous quotation emphasised human resources availability, the following quotation demonstrated a concern with the quality of primary mental health education when the interviewee described the relevance of MHMS to improve community health workers’ capacity to deal with mental health problems, mainly because they are considered gatekeepers of the health system.

*“MHMS have been qualifying community health workers. MHMS organised training sessions for their work (*mentioning community health workers*)”. (Health professional 1, FHS, GP)*

Furthermore, as well as showing an improvement in the capacity to identify and treat mental health problems, the next quotation underlines that FHS teams should understand, in technical terms, the primary mental health professional’s conceptual references.

*(…) but I do not know if people know that we need a methodology concerning MHMS teams’ references, something more formal, a structured methodology, (…). (health professional 2. FHS, GP).*

The Primary Mental Health Intervention category is concerned with how mental health actions in primary care have been delivered. The actions mentioned were: MHMS, support for the FHS team, prevention and mental health promotion, implementation of community therapy, home visits and joint consultations.

Positive evaluations included a clinical perspective of the primary care professionals when referring to the consequences of the integration.

*“I think that we have been improving diagnosis and treatment of mental illnesses”. (Health Professional, FHS, GP2)*

Moreover, primary mental health care interventions were considered important as professionals search for better understanding and to act closer to the patients’ context than was done in a traditional specialised mental health approach, as it usually focused on the individual’s disease only.

*“Mental health patients should be cared for in their area, with people they trust, in their home environment, in their territory”. (Health Professional, FHS, GP1)*

From a negative perspective, the general timetable was the main issue.

*“The problem with the matrix support is it takes place once a month, when we have many more patients that must be treated more frequently”. (Manager, FHS, nurse)*

This quotation not only demonstrates difficulties related to the integration of different work processes but also emphasises a concern with the continuity of mental health care, which is one of MHMS teams’ biggest challenges.

The table below summarises the main qualitative data findings, showing positive aspects as benefits and negative aspects as barriers to integrating mental health and primary care (Table 5).

**Table 5:** Open-ended question summary

Mental Health and Primary Care Integration	Positive aspects	Negative aspects
Network category	• Connecting primary care and mental health services. • Planning care together	• Lack of knowledge about different units, system structures and work processes • Constraints regarding institutional processes
Primary Mental Health education category	• Helping non-specialists managing mental health problems • Training SUS gatekeepers to identify mental health problems • Narrowing the communication gap between different work processes • Favouring primary teams to diagnose and prescribe appropriate interventions.	• Lack of human resources, excessive turnover of GPs, excessive working hours • Lack of knowledge regarding psychosocial interventions in PC
Primary Mental Health Interventions category	• Improving access to cases of difficult adherence • Delivering mental health care in the community • Integrating actions to care of co-morbidities	• Integrating different professionals’ timetables • Low frequency of mental health matrix support in the community (once a month) • Continuity of care is not perceived as a health tenet

### Mixed-method results

Faced with these results and using the 3 key issues in a framework, this study linked crucial points between the methods. Thereby, the outcomes were:

i) What are the health professionals’ perceptions about MHMS in Rio during the period studied?

The quantitative results showed that the professionals interviewed have been evaluating their actions only based on recent experiences or discussions. The qualitative results demonstrated that the workforce’s experiences pointed to challenges and difficulties to integrate different work processes, considering perceptions and experiences without clear and structured references.

ii) What are the similarities and differences between health managers’ and professionals’ perceptions?

While quantitative data showed that health managers and professionals had different perspectives only of the record system of this evaluation, qualitative data demonstrated the importance for both groups of clear communication, good relationships with other institutions and continuing primary mental health education to create a network and integrate actions from different perspectives. This shows how difficult it can be to integrate different work processes in a context where communication is problematic.

iii) What prospects are there for Brazilian MHMS?

Quantitative data about health managers and professionals demonstrated that these professionals converge in most of their perceptions. However, quantitative data about managers’ perceptions of service design contrasted different perspectives from traditional services and community-based services. The qualitative results emphasise how important it is to consider institutional designs to foster integration between different services without support or specific knowledge.

## Discussion

Evaluating primary care and mental health integration was planned as a local demonstrative project, exploring the perceptions of professionals involved in integrated care to offer primary mental health care to a low-income population, especially in community-based contexts. The main findings were:

1. The need for the creation of an instrument to evaluate the perception of professionals involved in PC and MH integration actions. The points of the questionnaire referred to the structure of the health system, the method of work between the professionals and the method of work to offer primary mental health care.
2. Health managers’ and professionals’ perceptions tend to agree in all points studied, except for Health Record System.
3. Managers’ perceptions of service design tend to disagree on points such as Access, Mental Health Interventions, Team Collaboration, Family Focus and Primary Mental Health Education. These perceptions tend to agree in terms of Gateway, Trust, Record System and Integration with Community Resources and Primary Mental Health Education.
4. A lack of communication and clear common knowledge about MHMS were underscored as crucial problems.
5. The mixed method demonstrated how important it is to contrast information concerning workforce and institutional perceptions faced with positive and negative MHMS aspects.
6. Health managers and professionals consider mental health and primary care integration as a requirement of the system.

These findings confirmed that planning and managing the integration of different work processes are fundamental to understanding the workforce’s point of view as well as to understanding they are strongly influenced by the model of work produced by the institutional culture [58]. For instance, these professionals value Primary Mental Health Education (3.4/5), and they are looking forward to improving their skills and knowledge to deliver mental health actions in Rio de Janeiro’s favelas, as the Primary Mental Health category highlighted. However, the workforce interviewed demonstrated awareness of constraints in institutional processes, human resources and knowledge [59]. These negative aspects suggest that despite health professionals’ being open and agreeing to work with MHMS, infrastructure problems have been identified in this vulnerable, low-income [60] and violent area, revealing inconsistencies between workforce and institutional perspectives.

Regarding the MHGAP, there was an interesting comparison of this setting with research in countries undergoing socio-economic conflicts and also wars in LAMIC. The literature review showed that although it is possible to facilitate primary care and mental health integration [61], indicators such as governance and different workforces [62] are better managed regionally [63], in the same health level platforms as in national perspectives, with different levels of health involved. Moreover, primary mental health care is, in some cases, considered a humanitarian solution, delivering health care to vulnerable people [64].

Nowadays, similar experiences of MHMS, as in Rio de Janeiro’s favelas, are growing in many Brazilian cities as a political strategy to improve the capacity of FHS teams to treat and prevent health problems and promote community-based health. This nationwide perspective has been implemented through Multidisciplinary Matrix support teams, where the presence of a mental health professional is always guaranteed [24]. This is especially important considering that Brazil is a country with more than 5000 municipalities, where this process is being massively implemented. However, as the findings emphasised, two different primary care platforms and a lack of clear information revealed a lack of common and clear objectives shared with this workforce [58].

For example, the evidence about the information recorders not only highlighted the relevance of an integrated information system but also these perceptions showed the concerns of these health professionals about how their work can be measured.

Another interesting example of a difference in viewpoints was that community-based health managers (PCC and FHS) evaluated better the sections on the community-based method of work, whereas the health managers working in traditional services evaluated them as worse. In order to integrate primary care and mental health it is important to recognise whether or not the MHMS purposes are coherent with health service culture to unite two different work processes. Hence, the need to create a programme integrating mental health into primary care in order to deliver primary mental health care and overcome the MHgap [27, 65].

These findings offer an insight into how primary mental health teams have experienced MHMS, suggesting that now is the time to qualify these actions [14], empower the workforce [5] and improve the network’s capability [66].

Likewise, especially noteworthy was the gap between work processes and expertise for the development of MHMS. These outcomes confirm the existence of subjective barriers in institutional relationships [23] and suggest that lack of communication might be responsible for points of tension between those involved, such as technical and ideological differences.

For example, in severe mental health cases, the collaboration between PCC and primary care services only happens when PCC patients need it and not when community-based primary care services identify the need of specialised collaboration in its area. This point may underline how important the link is between the need for institutional support and improved communication [14, 22, 67, 68].

Thus, the data identified some differences between health professionals as points of tension. For instance, even when the professional has a positive experience with MHMS, the qualitative data emphasised how difficult it is to combine two different backgrounds, sharing knowledge and making decisions together.

For further initiatives, explanatory guidelines, websites and telehealth might be important as a form of professional health education. Moreover, the creation of specific national primary mental health care indicators to evaluate these health services and practices is important to organise the primary mental health agenda [66], particularly considering the continuity of mental health care in primary care [69].

Some strengths of this study were:

- Contributing to the literature about primary care and community-based mental health integration in Brazil, Latin America and LAMIC;
- Integrating quali-quanti data such as health managers’ and professionals’ perceptions, health managers’ perceptions of different services involved in PC and MH and MHMS opinions in a mixed-methods study;
- Emphasising the association of health professionals’ workforce and institutional designs in public health as a fundamental issue to think about health services and research.

The limitations were:

- The number of interviewees was small, which suggests the need for further studies with a similar methodology in other areas of the city and the country;
- Data collection was done in two different periods. It would have been preferable to collect it all in the same period and a few questionnaire questions lack specificity in terms of mental health references or primary care;
- We did not interview important members of FHS teams such as nurses and community workers. This study also did not sufficiently evaluate long-term outcomes and was primarily aimed at doctors (GPs, psychiatrists and psychologists).

## Conclusion

The aim of this study was to map health managers’ and professionals’ perceptions about Primary Care and Mental Health by MHMS in Rio de Janeiro. The lessons learnt were especially about how to plan actions to deliver primary mental health care in vulnerable communities.

Although SUS is unified, this system has two primary care platforms in activity. The complexity of this background along with the findings concerning the record system suggests it is essential to integrate and record the available information about the integration process in a common health record system. This clearly demonstrates the reach and impact of primary mental health actions in the communities and how they can differ from traditional services.

These perceptions provided valuable lessons for policy planning and research, including aspects related to those points of tension where different perspectives on mental health care must be integrated. This involves engaging the policy makers, managers, primary care and mental health professionals on the ground. Hence, the study pointed to the challenge of connecting different health knowledge and the importance of communication strategies.

Additionally, health professionals clearly recognised the need to improve their knowledge about primary mental health care, underlining willingness on their part to rethink their actions. However, they need institutional support and motivation to be involved in further actions.

Concluding, in order to push the boundaries of traditional mental health interventions as a means of delivering mental health care in the community, not only did this study reveal that enhancing institutional communication is essential to integrate different work processes but it also shows that health professionals should be turned into stakeholders.

## Additional file

Additional file 1: Questionnaire Mapping the Integration of Mental Health Interventions in Primary Care. This questionnaire was originally in Portuguese. The English version was based on the original, considering only the items discussed in this original paper. (DOC 180 kb)

## Abbreviations

FHS: Family Health Strategy
GTO: General Traditional Outpatient
HDI: Human Development Index
LAMIC: Low and middle income countries
MH: Mental Health
MHGAP: Mental Health Gap
MHMS: Mental Health Matrix Support
MHOS: Mental Health Outpatient Services
PC: Primary Care
PCC: Psychosocial Community Centre
SDI: Social Development Index
SUS: Unified Health System
WHO: World Health Organization

## Footnotes

1 From this explanation on referred to the Brazilian National Health Service simply as SUS.

2 Grupo Interdisciplinar de Pesquisa em Atenção Primária à Saúde—CNPq (Conselho Nacional de Desenvolvimento Científico) (http://dgp.cnpq.br/dgp/espelhogrupo/9879901048555185).
