# Supplementary material for: Perceptions of health managers and professionals about mental health and primary care integration in Rio de Janeiro: a mixed methods study
Source: BMC Health Serv Res. 2016 Sep 30;16:532. doi: 10.1186/s12913-016-1740-8 (PMC5045579; doi:10.1186/s12913-016-1740-8)
Supplement: Additional file 1: — Questionnaire Mapping the Integration of Mental Health Interventions in Primary Care. This questionnaire was originally in Portuguese. The English version was based on the original, considering only the items discussed in this original paper. (DOC 180 kb) [file 12913_2016_1740_MOESM1_ESM.doc]

**Questionnaire Mapping the Integration of Mental Health Interventions in Primary Care**

Based on the questionnaires elaborated by:

1. Barbara Starfield, MD, MPH, FRCGP & James Macinko, PhD, Dept. of Health Policy & Management, Johns Hopkins Bloomberg School of Public Health, Baltimore, MD USA
2. James Macinko (New York University) & Celia Almeida (ENSP/FIOCRUZ). Questionnaire about the Primary Health Care System (Sistema de Atenção Básica) in Brazil, 2005.
3. Stella R. Taquette. A tool put together by a multidisciplinary team made up of GPs, psychologists, social workers and epidemiologists in a research project approved in the Edital da Faperj "Prioridade Rio" 2010.

**ATHIÉ, K; FORTES, S. Questionnaire Mapping the Integration of Mental Health Care Interventions in Primary Care, 2011.**

| **GENERAL INFORMATION** (to map profession, position and workplace) descriptive answers  Descritivas | |
| --- | --- |
| 1 | Date |
| 2 | Interviewer’s Name |
| 3 | Interviewee’s Name |
| 4 | Interviewee’s Profession |
| 5 | Interviewee’s Position |
| 6 | Number of years working in this position |
| 7 | Unit |
| 8 | Address |
| 9 | City |
| 10 | Opening hours |
| 11 | Telephone Number |
| 12 | Fax Number |
| 13 | Type of Organisation  a. General Outpatients ( )  b. Mental Health Specialised Outpatients ( )  c. CAPS Psychosocial Community Centre ( )  f. FHS Family Health Strategy ( ) |
|
|
|
|

**The answers to the questions of each of the topics described below are closed. Please state how often they occur in your health unit.**

| **ACCESS** (To map whether patients can access services, medicines and consultations). For the item consider the following answers / frequencies to the question: 0 for DON’T KNOW; 1 for NEVER (0%); 2 for ALMOST NEVER (1-25%); 3 for SOMETIMES (26-50%); 4. for ALMOST ALWAYS (51-75%); 5. for ALWAYS (76-100%); 88 when the question does not apply to the kind of organisation being referred to.  (*) **Adequate means**: every day all medicines (or equipment) are available to meet the clients’ / users’ needs (not urgent or specialized), as they seek treatment in this unit. | |
| --- | --- |
| 1 | In the last 6 months has there been an adequate offer of essential medicines for chronic mental health problems? |
| 2 | Does the unit open on Saturdays? |
| 3 | During the week does the unit stay open after 6pm at least once a week? |
| 4 | During the unit’s opening hours, is there a telephone number to make appointments? |
| 5 | During the unit’s opening hours, is there a telephone number to ask for information? |
| 6 | Does the user wait for more than 60 minutes in the unit before being seen by a health professional? |
| 7 | When a patient gives up treatment, is there an active search? |
| 8 | When the patient misses their appointment, is it filled by another patient? |
| 9 | Are the services available in your unit made public in schools? (every 6 months) |
| 10 | Are the services available in your unit made public in neighborhood associations? |
| 11 | Are the services available in your unit made public in NGOs? |
| 12 | Are the services available in your unit made public in your region’s healthcare network? |

| **GATEWAY** (To map the different services available to patients in the unit). For the item consider the following answers / frequencies to the question: 0 for DON’T KNOW; 1 for NEVER (0%); 2 for ALMOST NEVER (1-25%); 3 for SOMETIMES (26-50%); 4. for ALMOST ALWAYS (51-75%); 5. for ALWAYS (76-100%); 88 when the question does not apply to the kind of organisation being referred to. | |
| --- | --- |
| 1 | Before consulting a specialist, for the user to be seen in their unit, is a GP consulted? (Except in cases of emergency) |
| 2 | Is the individual received on the day the appointment is made? |
| 3 | Is there a reception group? |
| 4 | Is there a family reception? |
| 5 | Is enrolment in your unit on demand? (When the unit’s resources correspond to the patient’s needs and not in any case) |
| 6 | Is enrolment in your unit by formal referral? |
| 7 | Is enrolment in your unit by catchment area? |
| 8 | Is enrolment in your unit by registration? |

| **TRUST** (To map the relationship between patients and health professionals, and patients and units). For the item consider the following answers / frequencies to the question: 0 for DON’T KNOW; 1 for NEVER (0%); 2 for ALMOST NEVER (1-25%); 3 for SOMETIMES (26-50%); 4. for ALMOST ALWAYS (51-75%); 5. for ALWAYS (76-100%); 88 when the question does not apply to the kind of organisation being referred to. | |
| --- | --- |
| 1 | How often are users / clients seen by the same professional when consulted? |
| 2 | How often are users / clients seen by the same team when consulted? |
| 3 | Does the unit have a population in a catchment area? |
| 4 | If the user has a doubt about treatment, can they phone to speak with the same professional that saw them? |
| 5 | Do you think the health professionals in your unit give clients enough time to explain fully their doubts and worries? |
| 6 | Do the unit’s health professionals use the medical record every consultation? |
| 7 | How often are the health professionals informed about all the medicines used by the user / client during their consultation? |
| 8 | How often are the health professionals informed about the user’s / client’s therapeutic project during their consultation? |
| 9 | Are health professionals informed when a user cannot obtain / buy the medicine prescribed? |
| 10 | Does the professional see the patient periodically even without spontaneous demand? (longitudinality / continuity of care) |
| 11 | How often do users know the name of the professionals who see them in the unit? |
| 12 | How often do users recognise the professionals that see them in the unit? |

| **PRIMARY MENTAL HEALTH INTERVENTIONS** (To map psychosocial actions offered). For the item consider the following answers / frequencies to the question: 0 for DON’T KNOW; 1 for NEVER (0%); 2 for ALMOST NEVER (1-25%); 3 for SOMETIMES (26-50%); 4. for ALMOST ALWAYS (51-75%); 5. for ALWAYS (76-100%); 88 when the question does not apply to the kind of organisation being referred to. | |
| --- | --- |
| 1 | Individual reception (at the time the appointment is made) |
| 2 | Group reception |
| 3 | Family approach |
| 4 | Waiting room group |
| 5 | Individual psychological consultation |
| 6 | Consultation with occupational therapist |
| 7 | Joint consultation with mental health professional |
| 8 | Nursing consultation |
| 9 | Psychiatric consultation |
| 10 | Consultation with a social worker |
| 11 | Treatment of mental health cases by the GP |
| 12 | Consultation with a neurologista |
| 13 | Interconsultations and discussion of cases with a mental health professional |
| 14 | Multidisciplinary meeting |
| 15 | Matrix support (external – when the action needs to occur between different teams and there is a need to visit an external unit) |
| 16 | Supervision |
| 17 | Institutional visit |
| 18 | Worker’s mental health |
| 19 | Complementary therapies (acupuncture, auriculotherapy) |
| 20 | Meeting with the community |
| 21 | External meeting (with the aim of strengthening the integration between the mental healthcare network and other units) |
| 22 | Therapeutic Groups in Mental Healthcare (conducted by specialists) |
| 23 | Therapeutic Groups in Mental Healthcare (non-specialists) |
| 24 | Home visit |
| 25 | Prescription of psychotropics |
| 26 | Free dispensation of psycotropics |
| 27 | Workshops (groups with specific activities such as handicraft, dance, theatre, painting) |
| 28 | General meeting (activities bringing together professionals and users to discuss the services) |
| 29 | Active search |
| 30 | Others |
|  | Please specify: |

| **PRIMARY MENTAL HEALTH RECORD (**To map if mental health interventions are recorded in the Health Record System as mental health interventions or primary care interventions). For the item consider the following answers / frequencies to the question: 0 for DON’T KNOW; 1 for NEVER (0%); 2 for ALMOST NEVER (1-25%); 3 for SOMETIMES (26-50%); 4. for ALMOST ALWAYS (51-75%); 5. for ALWAYS (76-100%); 88 when the question does not apply to the kind of organisation being referred to. | |
| --- | --- |
| 1 | | Does the information system have specific records for the kinds of healthcare services that you receive? | | --- | |
| 2 | As for the above-mentioned interventions, is mental health care assistance recorded? |
| 3 | Is the information system adequate for the invoicing of the activities carried out? |

| **COLLABORATION BETWEEN TEAMS.** For the item consider the following answers / frequencies to the question: 0 for DON’T KNOW; 1 for NEVER (0%); 2 for ALMOST NEVER (1-25%); 3 for SOMETIMES (26-50%); 4. for ALMOST ALWAYS (51-75%); 5. for ALWAYS (76-100%); 88 when the question does not apply to the kind of organisation being referred to.  (***Matrix support**: collaboration between the mental healthcare professional and the GP)  **(*Therapeutic Project :** a commonly-used expression in health services referring to the care plan put together by the healthcare team or professional)  (To map collaborative work with different health teams and services as well as with other institutions such as health services, schools or community services.) | |
| --- | --- |
| 1 | In your team, do you consider that mental health interventions are carried out? |
| 2 | Does the team meet to discuss/elaborate individual therapeutic projects? |
| 3 | In your unit, does the team meet to discuss the group / family therapeutic projects? |
| 4 | Does the team meet to discuss/elaborate therapeutic projects aimed at community interventions? |
| 5 | In your unit, when a professional refers a case, is there a reply to the counter-referral? |
| 6 | How often do non-specialised mental healthcare professionals of your health unit take care of patients with problems considered to be related to mental health? |
| 7 | How often do non-specialised mental healthcare professionals of your health unit seek solutions to these problems by liaising with mental healthcare professionals? |
| 8 | How often do professionals of your health unit refer the mental healthcare user to mental healthcare specialists? |
| 9 | Does the team use collaborative work to integrate the above-mentioned interventions? |
| 10 | Does the team ask for the help of other professionals who work in other units to carry out the collaborative work? |
| 11 | Does the team receive these professionals well to carry out the collaborative work when it happens in their unit? |
| 12 | Does the team seek the other professionals’ help to carry out the collaborative work outside the unit? |
| 13 | Does the team collaborate with other kinds of health arrangements to integrate the above-mentioned interventions? |
| A1/B1/ C1 | Which other kinds of arrangements? |
| A2/B2/ C2 | Please specify: |

| **MENTAL HEALTH PROBLEMS** (To map mental health problems treated in the unit). Regarding mental health problems, how often do you observe users’ presenting problem in your unit. For the item consider the following answers / frequencies to the question: 0 for NONE; 1 for LOW (1-30%); 2 for MODERATE (31-60%); 3 for HIGH (61-100%); 99 Don’t know. | |
| --- | --- |
| 1 | Anxiety |
| 2 | Depression |
| 3 | Alcohol abuse |
| 4 | Alcoholism |
| **Drug Use** | |
| 5.a | Use of Crack |
| 5.b | Cocaine |
| 5.c | Cannabis |
| 6 | Personality disorders |
| 7 | Family conflicts |
| 8 | Somatisation |
| 9 | Learning problems |
| 10 | Domestic violence |
| 11 | Psychosis |
| **Serious Mental Disorders stemming from Childhood and Adolescence** | |
| 12.a | Retardedness |
| 12.b | Autism |
| 12.c | Behaviour disorder |
| 13 | Dementia |
| 14 | Post-traumatic stress disorders |
| 15 | Medication abuse |
| 16 | Sexual abuse |
| 17 | Sexual dysfunction |

| **FAMILY FOCUS** To map family interventions. For the item consider the following answers / frequencies to the question: 0 for DON’T KNOW; 1 for NEVER (0%); 2 for ALMOST NEVER (1-25%); 3 for SOMETIMES (26-50%); 4. for ALMOST ALWAYS (51-75%); 5. for ALWAYS (76-100%); 88 when the question does not apply to the kind of organisation being referred to. | |
| --- | --- |
| 1 | Is the organisation of the medical records by family? |
| 2 | During consultations, do the healthcare professionals normally ask about other members of the family’s diseases? |
| 3 | Are consultations carried out in the family’s presence with the focus on individuals’ health in relation to their family situation? |
| 4 | Is the genogram or the familiogram a resource used during consultations? |
| 5 | During the anamnesis, do healthcare professionals ask about social risk factors or the user’s living conditions? (unemployment, living arrangements, family situation)? |
| 6 | In your unit, is there a trained professional whose care focus is the family? |

| **INTEGRATION WITH COMMUNITY RESOURCES** Regarding frequency of integration of your unit with the community, which community resources do you identify as partners? (To map institutional integration with community resources). For the item consider the following answers / frequencies to the question: 0 for DON’T KNOW; 1 for NEVER (0%); 2 for ALMOST NEVER (1-25%); 3 for SOMETIMES (26-50%); 4. for ALMOST ALWAYS (51-75%); 5. for ALWAYS (76-100%); 88 when the question does not apply to the kind of organisation being referred to. | |
| --- | --- |
| 1 | General Outpatients |
| 2 | Family Health Strategy |
| 3 | Psychosocial Community Centre |
| 4 | NGOs |
| 5 | Social Centre |
| 6 | Religious Institution |
| 7 | School |
| 8 | Neighbourhood Association |
| 9 | Samba School |
| 10 | Other |

| **PRIMARY MENTAL HEALTH EDUCATION** (To map educational expectations regarding mental health in primary care.). For the item consider the following answers / frequencies to the question: 0 for DON’T KNOW; 1 for NEVER (0%); 2 for ALMOST NEVER (1-25%); 3 for SOMETIMES (26-50%); 4. for ALMOST ALWAYS (51-75%); 5. for ALWAYS (76-100%); 88 when the question does not apply to the kind of organisation being referred to. | |
| --- | --- |
| 1 | Do you think that the professionals in your unit are interested in participating in permanent education activities in mental health? |
| 2 | Does your unit promote internally permanent education activities for its professionals? |
| 3 | Does the town council offer permanent education activities in mental health for professionals in your unit? |

| **OPEN-ENDED QUESTION** |
| --- |
| Considering the perspective of mental health matrix support, in your opinion, what are the positive and negative points of integration between mental health and primary care in the region where you work (Programmatic Area)? |
|  |

N.B.: This questionnaire was originally in Portuguese. The English version was based on the original, considering only the items discussed in this original paper.
